# Profiles of calreticulin and Ca^2+^ concentration under low temperature and salinity stress in the mud crab, *Scylla paramamosain*

**DOI:** 10.1371/journal.pone.0220405

**Published:** 2019-07-25

**Authors:** Huiyang Huang, Chencui Huang, Limei Guo, Chaoshu Zeng, Haihui Ye

**Affiliations:** 1 College of Ocean and Earth Sciences, Xiamen University, Xiamen, China; 2 College of Science and Engineering, James Cook University, Townsville, Queensland, Australia; Shanghai Ocean University, CHINA

## Abstract

Calreticulin (CRT) is an important molecular chaperon crucial to survival of organisms under adverse conditions. In this study, the potential roles of CRT in the mud crab, *Scylla paramamosain*, were investigated. Firstly, *SpCRT* gene expression was detected in various tissues of *S*. *paramamosain* with the highest expression found in the hepatopancreas. To evaluate potential role of *SpCRT* in cold adaption, sub-adult crabs were subjected to temperatures of 10, 15, 20 and 25°C and the profiles of *SpCRT* gene were determined in the hepatopancreas, chela muscle and gills. The results showed that the expressions of *SpCRT* mRNA in these tissues were significantly higher for those crabs exposed to low temperatures of 10 and 15°C as compared to those exposed to the higher temperatures, indicating *SpCRT* was involved in cold adaptation—probably through facilitating protein folding. When low temperature 10°C or 15°C was further combined with high and low salinity stress, the expression of *SpCRT* mRNA at low salinity (10 ppt) was in most cases significantly higher than that at high salinity (35 ppt), suggesting that under low temperatures, low salinity may represents a more stressful condition to the crab than high salinity. It was also shown that when crabs challenged by 10°C, Ca^2+^ concentration increased rapidly in the hepatopancreas and an *in vitro* experiment further showed that the expression of *SpCRT* mRNA increased concurrently with added Ca^2+^ concentration; these results together imply that Ca^2+^ probably plays a major role in low temperature signaling, which induces expression of genes related to cold adaption, such as *CRT*.

## Introduction

Calreticulin (CRT) is an endoplasmic reticulum (ER) luminal resident protein and ubiquitously expressed in all of multicellular eukaryotes. CRT is comprised of three highly conserved domains: N-domain, a protein-interacting domain; P-domain, a Proline-rich domain; and C-domains, which are Ca^2+^-interacting domains [1,2]. Recent studies have shown that CRT plays key roles in eukaryotic Ca^2+^ homeostasis, Ca^2+^-dependent signal pathways and molecular chaperoning [3–5]. CRT is therefore involved in many biological processes, ranging from growth, development, reproduction, molting, immune systems, stress responses and cellular defence [1,6–8]. In crustaceans, CRT is known to play important roles in molting, immune functions and stress responses [9,10], moreover, studies showed that CRT is one of the most important elements for low temperature and salinity stress in many species [11–15].

The mud crab, *Scylla paramamosain*, is widely distributed in intertidal and subtidal zones in the coasts of southeast China, and is a commercially important species for both fisheries and aquaculture [16,17]. Although the crab species can survive at temperatures between 7–37°C, the optimum temperature for growth and development are between 18–30°C [18]. The biological zero, temperature at which embryos cannot complete their development, is 12.19°C in *S*. *paramamosain* [19]. In fact, cold snaps in winter can bring about mass mortality of this species in aquaculture in subtropics. Due to its important economic status, more and more researches have been done on the physiological adaptability of the mud crab to low temperature effect [20,21]. Several recent studies have showed that CRT is closely related to a number of stress responses, including oxidation, starvation, virus infection, heat shock and salinity stresses [15,22–25]; however, there is a lack of study focusing on function of CRT on low temperature adaption.

In this study, we obtained the *CRT* in *S*. *paramamosain* (*SpCRT*) from GenBank (GenBank accession no. HQ260918) and profiled its expression levels in different tissues. To further understand the physiological processes involving in low temperature adaption in *S*. *paramamosain*, the effects of low temperature stress on *SpCRT* expression were examined by quantitative real-time PCR. Since temperature and salinity often affect the wellbeing of marine species in concert, we further subjected *S*. *paramamosain* to low temperatures in combination with different salinities to examine their effects on *SpCRT* expression. In addition, the relationship of Ca^2+^ concentration and the expression of *SpCRT* gene under low temperature stress were evaluated.

## Material and methods

### Experimental animals

The mud crabs *S*. *paramamosain* were sourced commercially from Xiang’an District, Xiamen, Fujian Province, China. Three mature crabs with carapace length of 8.5 ± 0.6 cm and body weight of 370 ± 35 g were used to analysis of the tissue distribution of *SpCRT*. For the low temperature stress experiment, 300 sub-adult crabs (carapace length: 5.1 ± 0.7 cm; body weight: 90 ± 8 g) were used instead. All of the crabs in this study were healthy and with intact appendages. When the crabs arrived in the laboratory, they were firstly acclimated to common aquaculture conditions (temperature 25 ± 0.5°C; salinity 25 ppt) for two days, during which the crabs were fed live clam, *Ruditapes philippinarum*. The study does not involve endangered or protected species.

### Nucleic acid extraction and cDNA synthesis

Total RNA was isolated from each of the crab samples using RNAizol reagent (Invitrogen, USA) based on the manufacturer’s instruction. The RNA integrity and concentration were detected by 1.5% agarose gel electrophoresis and a NanoDrop 2000 spectrophotometer (NanoDrop Technologies, USA), respectively.

For first-strand cDNA synthesis, 2 μg total RNA was treated with RNase-free DNase I (TaKaRa, Japan) to remove genomic DNA, and then reversely transcribed by using the Revert Aid. First-strand cDNA Synthesis Kit (Fermentas) and random primers were used for cDNA synthesis.

### Quantitative real-time PCR analysis

Recent studies have showed that the housekeeping genes could be influenced by various factors, such as different tissues and development stages, leading to potential discrepancies of the results. In view of this, it is necessary to select two or three appropriate housekeeping genes for the analysis of gene expression [11,26,27]. According to the method previously described in *S*. *paramamosain* [27], the analysis was carried out on five replicates and normalized by the average of three housekeeping genes: 18S rRNA (GenBank ID: FJ774906), β-actin (GenBank ID: GU992421) and GAPDH (GenBank ID: JX268543).

The qRT-PCR was employed to detect *SpCRT* mRNA levels in following 10 different tissues of *S*. *paramamosain*: cerebral ganglion, eyestalk ganglion, heart, stomach, gill, hepatopancreas, thoracic ganglion, chela muscle, epidermis and hemocytes.

In the low temperatures stress experiment, *SpCRT* mRNA levels in the hepatopancreas, chela muscle and gills were detected by qRT-PCR for crabs exposed to different temperatures. Gene-specific primers (Table 1) were designed to amplify a 101 bp fragment of coding sequence. The reaction mixture of 20 μl in total contained 10 μl of 2 × SYBR Premix Ex Taq (TaKaRa, Japan), 1.2 μl of diluted cDNA template, 0.8 μl of each 10 μM primers, and 6.4 μl of ddH_2_O. Real-time PCR conditions were 95°C for 10 min, 40 cycles of 95°C for 20 s, 54°C for 30 s, 72°C for 30 s. The gene expression levels were calculated by the 2^-ΔΔCt^ comparative threshold cycle (Ct) method.

**Table 1:** Primers used in cloning and characterizing the gene of *SpCRT*.

Name	Sequence (5′–3′)	PCR objective
SpCRT-F3	CTATGGCCTGTCCAGCAA	Real-time PCR
SpCRT-R3	CAAACATGACAAGGTAGGGA	Real-time PCR
18S rRNA-F	CAGACAAATCGCTCCACCAAC	Internal control
18S rRNA-R	GACTCAACACGGGGAACCTCA	Internal control
GAPDH-F	AATGCCATCACAATAGAAAAATC	Internal control
GAPDH-R	GGAACAATCAACACTACCACACC	Internal control
β-actin-F	GAGCGAGAAATCGTTCGTGAC	Internal control
β-actin-R	GGAAGGAAGGCTGGAAGAGAG	Internal control

### Temperatures stress experiment

Five temperature conditions, 5, 10, 15, 20 and 25°C, were set up using temperature-controlled incubators, and the sub-adult crabs were randomly allocated into five incubators. All crabs were placed individually in round plastic containers (diameter 10 cm × height 12 cm) filled to about one third of volume with filtered seawater of salinity 25 ppt. At 0 h immediately prior to the crabs being transferred to colder temperatures, samples of 5 crabs acclimated to 25°C were taken as the control. Then at 1, 3, 6, 12, 24 and 48 h of exposure to cooler temperatures, five crabs were randomly sampled from each of the five temperature treatments, and the hepatopancreas, chela muscle and gills were dissected out. These tissues were frozen immediately in liquid nitrogen and stored in -80°C for later use.

### Low temperatures in combinations with salinity stress experiment

Sub-adult crabs were exposed to four combinations of two low temperatures (10 ± 0.2°C and 15 ± 0.2°C) with a low (10 ± 0.1) or a high salinity (35 ppt ± 0.1 ppt) to investigate their effects on expression of *SpCRT*. To obtain the desired salinities, natural seawater (salinity of 25 ppt) was firstly adjusted to be slightly higher than salinity of 35 ppt by adding marine salt, and then diluted with distilled water to salinity of 35 ppt and 10 ppt, respectively. Other experimental procedures were the same as the temperatures stress experiment described above.

### Total calcium level in the hepatopancreas

Based on the result from the temperature experiment that the expression of *SpCRT* mRNA was the highest at 10°C in the hepatopancreas, 10°C was chosen for the further research on effects of low temperature stress on Ca^2+^ levels in the hepatopancreas.

Hepatopancreas samples for total calcium analysis were prepared using the method modified from Wilder et al. [28]. Briefly, samples were weighed and placed in a Petri dish for drying in an incubator at 110°C for at least 48 h until reaching constant weights. Approximately 1.0 g dry weight of each sample was mixed with 5 mL nitric acid and 2 mL of hydrogen peroxide and digested in a microwave oven (Multiwave 3000; Anton Paar GmbH, Stuttgart, Germany) for over 2 h. The digests were then diluted with deionized water and external calibration curves were constructed for determining the total Ca^2+^ levels. The concentration of calcium was measured using atomic absorption spectrophotometer (AA-6300C, Shimadzu, Japan) at the wavelengths of 422.7 nm.

### *In vitro* experiment: Effect of Ca^2+^ on *SpCRT* expression

Sub-adult crabs were sterilized in 75% ethanol after being immobilized on ice for 10 min. The hepatopancreas were dissected from the crabs and rinsed nine times with saline solution modified for crabs (hereafter referred to as ‘crab saline solution’): 440 mM NaCl, 11.3 mM KCl, 13.3 mM CaCl_2_, 26 mM MgCl_2_, 23 mM Na_2_SO_4_, 10 mM Hepes (pH 7.4), and contained penicillin G (300 IU/ml) and streptomycin (300 mg/ml, Sigma–Aldrich Chemical Co.). The hepatopancreas tissues were cut into small pieces of ~50 mg, each piece of the tissue was then placed in a well of a 24-well culture plate with 250 μl of medium L15 and 10 μl Ca^2+^ at one of the four following concentrations, 0, 5, 10 and 15 mM, added. The Ca^2+^ solutions were prepared beforehand with medium L15. Each of Ca^2+^ concentration treatment was triplicated and the culture plates were incubated at 26 ± 0.3°C for 12 h before the total RNAs from the tissues were extracted.

### Statistical analysis

The relative expression of *SpCRT* and relative concentration of Ca^2+^ were analyzed using one-way ANOVA followed by Duncan’s multiple range tests. All statistical analysis was performed on SPSS 16.0 software (SPSS Inc., Chicago, USA). Differences were considered as significance when *P* value < 0.05.

## Results

### Tissue distribution of *SpCRT*

**Fig 1:**
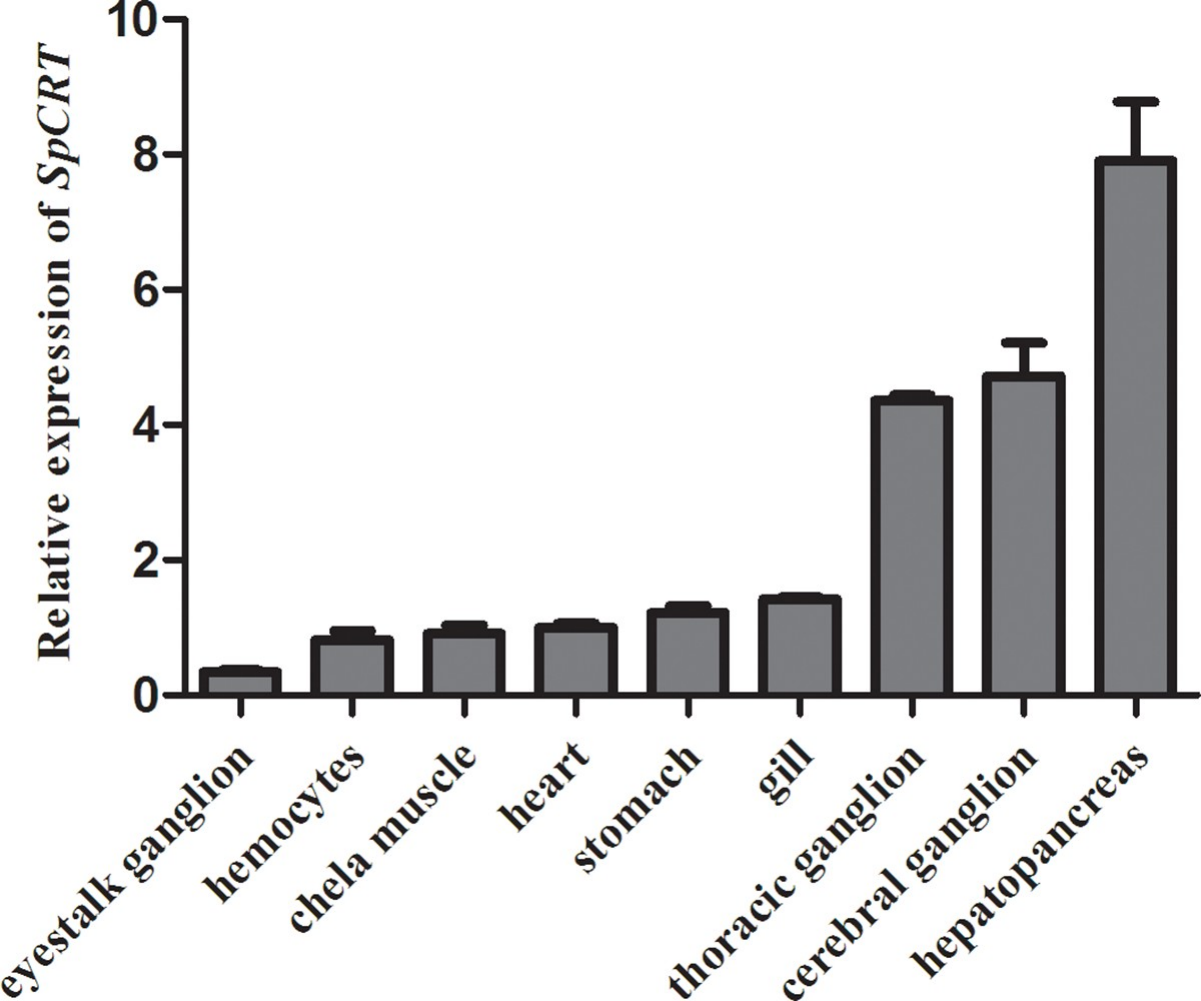
Tissue distribution of *SpCRT* in the mud crab, *S*. *paramamosain* by qRT-PCR. The relative expression of *SpCRT* is normalized by the average of three housekeeping genes: 18S rRNA (GenBank ID: FJ774906), β-actin (GenBank ID: GU992421) and GAPDH (GenBank ID: JX268543). Data are presented as mean ± standard deviation (n = 5).

Fig 1 shows the expression levels of *SpCRT* gene in 10 different tissues of *S*. *paramamosain*: cerebral ganglion, eyestalk ganglion, heart, stomach, gill, hepatopancreas, thoracic ganglion, chela muscle, epidermis and hemocytes. *SpCRT* mRNA was most abundantly expressed in the hepatopancreas, followed by that in the cerebral ganglion and thoracic ganglion.

### Expression levels of *SpCRT* mRNA in the hepatopancreas, chela muscle and gills in crabs subjected lower temperatures

When the mud crabs acclimated to the control temperature of 25°C were subjected to lower temperatures, they were observed to move normally at 20°C, but with substantially reduced activities at 15°C. At 10°C, they were largely un-active unless touched. At 5°C, the crabs quickly became motionless and were in a state of anesthesia, all of them eventually died within 12 h of cold stress. Hence no data was analyzed on *SpCRT* mRNA expression for the treatment.

**Fig 2:**
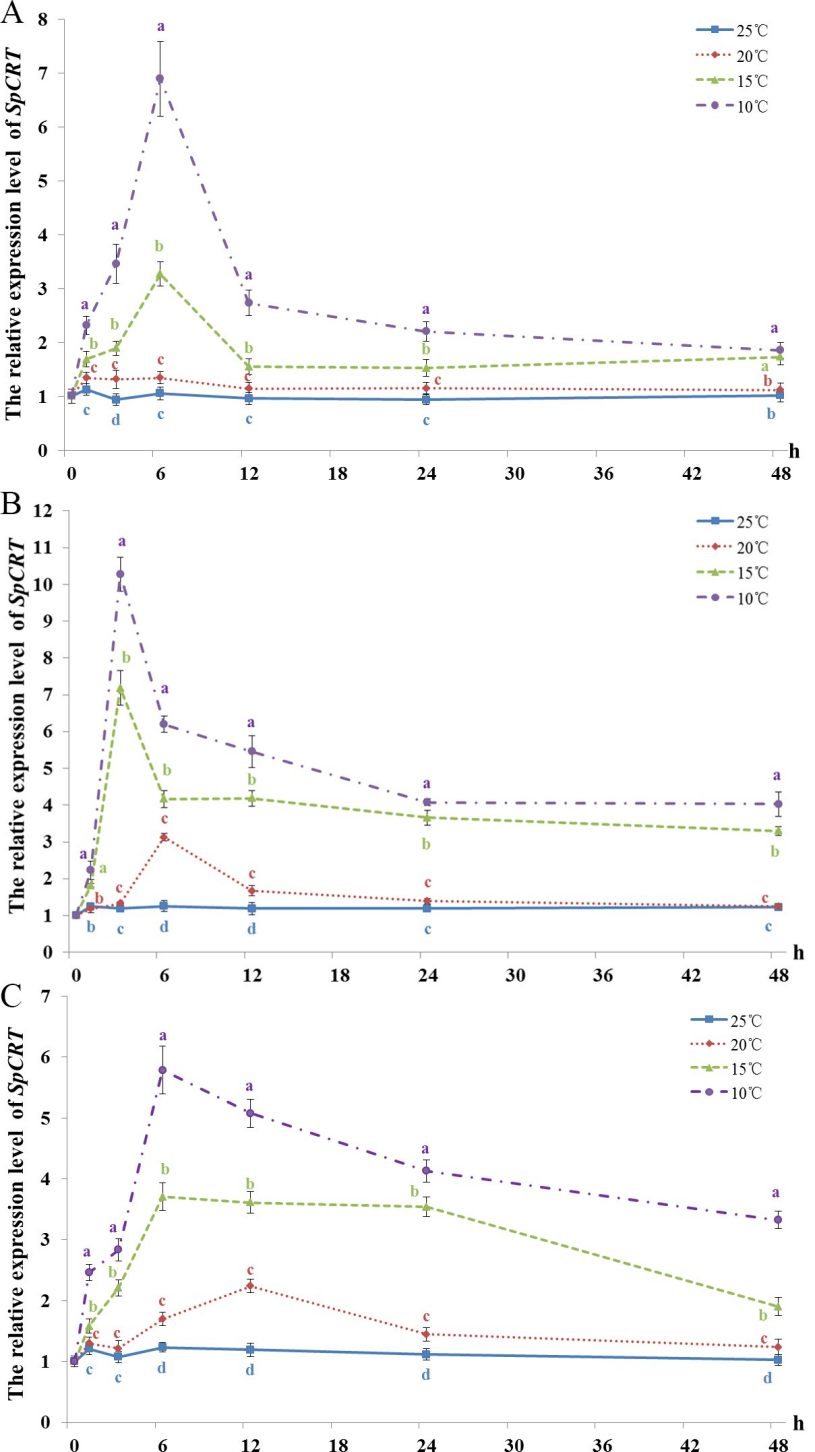
**The relative expressions of *SpCRT* in the hepatopancreas (A), chela muscle (B) and gill (C) of *S*. *paramamosain* subjected to different temperatures.** Data are presented as mean ± standard deviation (n = 5). Significant differences are indicated by different letters (*P*<0.05).

The expression levels of *SpCRT* mRNA in the hepatopancreas remained stable throughout the experiment duration at 25°C (Fig 2A). The expression levels of *SpCRT* mRNA at 20°C were similarly very stable throughout 48 h exposure and was consistently higher than those of 25°C although the differences were not significant (*P*>0.05). In contrast, when the crabs exposed to lower temperature of 15°C, the abundance of *SpCRT* mRNA increased sharply (*P*<0.05) from 0 h to 6 h before dropped back and remained at much lower levels than the peak value at 12 h and onwards. Moreover, the expression levels of *SpCRT* mRNA at 15°C were consistently significantly higher than those of 20°C and 25°C at all sampling times (*P*<0.05). The *SpCRT* mRNA expression pattern at 10°C was similar to that of 15°C, but were mostly significantly higher than those of 15°C (*P*<0.05) except at 24 h (*P*>0.05). In fact, the expression levels of *SpCRT* mRNA at 10°C were consistently the highest among all temperature treatments at each sampling point and the peak expression level at 6 h was almost 7-fold higher than that of the crabs acclimated to the control temperature of 25°C (0 h) (Fig 2A).

The expression levels of *SpCRT* mRNA in chela muscle again remained stable at 25°C (Fig 2B). The expression levels of *SpCRT* mRNA at 20°C were mostly similar to those of 25°C except at 6 h when increased sharply, leading to significantly higher levels than 25°C at 6 h and 12 h (*P*>0.05). At 15°C, *SpCRT* mRNA level rapidly and significantly up-regulated and reached a peak at 3 h after cold challenge, with the peak expression level more than 7-fold that of the crabs acclimated to 25°C (0 h); it dropped back to a lower level at 6 h and onwards but remained significantly higher than those at 20 and 25°C (*P*<0.05). The *SpCRT* mRNA expression pattern at 10°C was similar to that of 15°C with a peak expression at 3 h, which was more than 10-fold higher than that of the crabs acclimated to 25°C; it decreased sharply at 6 h and continued to gradually decrease till 24 h and remained stable afterward. At each sampling time, the expression levels of *SpCRT* mRNA at 10°C were significantly higher than those of all other treatments (*P*<0.05) (Fig 2B).

The expression of *SpCRT* mRNA in gills largely remained stable at 25°C. The expression of *SpCRT* mRNA at 20°C increased gradually and reached a peak at 12 h before showed a decreasing trend afterward; they were significantly higher than those at 25°C at most sampling points (*P*<0.05). Consistent significantly higher expression levels of the mRNA were observed at 15°C than at 20°C and 25°C (*P*<0.05) and the expression showed a pattern of increasing rapidly to reach maximum between 6 and 24 h before decreasing afterward. At 10°C, the *SpCRT* mRNA expression increased continuously to reach a peak at 6 h before decreasing gradually afterward. At each sampling time, the expression level at 10°C was significantly higher than any other treatments (*P*<0.05) (Fig 2C).

In summary, the expression of *SpCRT* mRNA in the hepatopancreas, chela muscle and gills showed a consistent pattern of lower temperatures induced significant higher expression levels. The *SpCRT* mRNA expression at 20°C induced mild up-regulating, while *SpCRT* mRNA was markedly up-regulated at 15°C and more so at 10°C, which induced significant higher expression levels than all three higher temperatures tested.

### Expression levels of *SpCRT* mRNA in the hepatopancreas, chela muscle and gills when crabs subjected to low temperatures combined with salinity stress

**Fig 3:**
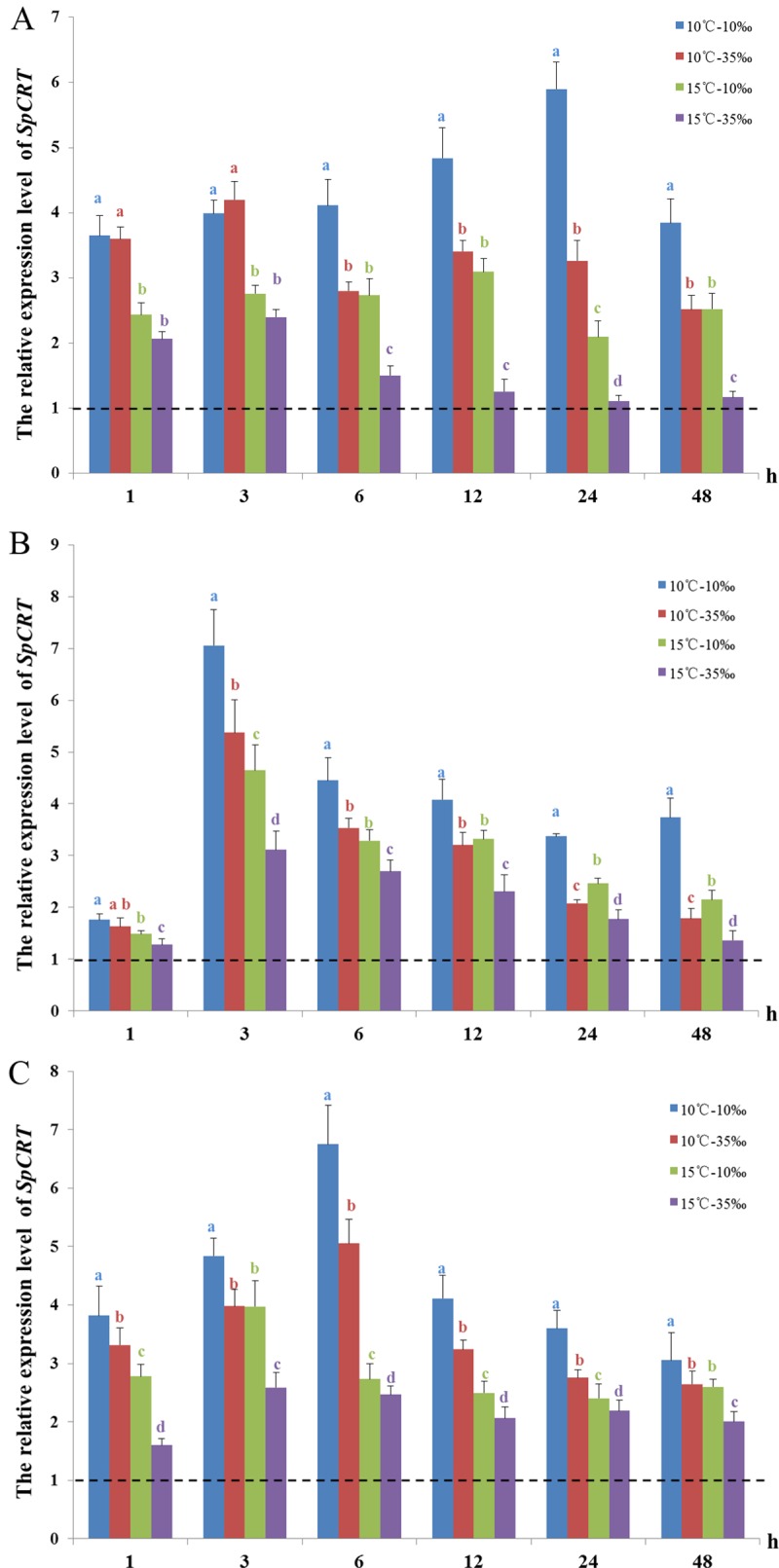
**The relative expression of *SpCRT* mRNA in the hepatopancreas (A), chela muscle (B) and gills (C) of *S*. *paramamosain* when subjected to combined low temperature and salinity stress.** The dotted line represents *SpCRT* expression in tissues of the crabs under the control condition (25°C; salinity 25ppt), corresponding to the value of 1 in the Y-axis. Data are presented as mean ± standard deviation (n = 5). Different letters on the top of histogram bars indicate significant differences (*P*<0.05).

Compared to the control (25°C-salinity 25 ppt), the expression of *SpCRT* mRNA in the hepatopancreas was up-regulated under each of our combined low temperature and salinity treatment conditions (Fig 3A). In particular, the maximum expression of *SpCRT* mRNA of the 10°C-salinty10 ppt, 10°C-salinity 35 ppt, 15°C-salinity 10 ppt and 15°C-salinity 35 ppt treatment was 5.9-fold, 4.2-fold, 3.1-fold and 2.4-fold higher than that of the control, respectively. Under a same low temperature (10°C or 15°C), the expression of *SpCRT* mRNA at low salinity 10 ppt was significantly higher than that at the high salinity 35 ppt from 6 h onward (*P*<0.05). On the other hand, under a same salinity (salinity 10 ppt or 35 ppt), the abundance of *SpCRT* mRNA at lower temperature 10°C was always dramatically higher than that at 15°C (*P*<0.05) (Fig 3A).

The expression of *SpCRT* mRNA in the chela muscle was similarly up-regulated in all four treatments as compared to the control, particularly from 3 h onwards (Fig 3B). The maximum expression of *SpCRT* mRNA at 10°C-salinity 10 ppt, 10°C-salinity 35 ppt, 15°C-salinity 10 ppt, 15°C-salinity 35 ppt was 7.1-fold, 5.4-fold, 4.6-fold and 3.1-fold higher than that of the control, respectively. Under a same low temperature (10°C or 15°C), the expression of *SpCRT* mRNA at salinity 10 ppt was significantly higher than that at 35 ppt from 3 h onwards (*P*<0.05) while under a same salinity (10 ppt or 35 ppt), the abundance of *SpCRT* mRNA at 10°C was always significantly higher than that at 15°C (*P*<0.05) (Fig 3B).

The expression of *SpCRT* mRNA in the gills was also up-regulated in all four treatments as compared to the control. The maximum expression of *SpCRT* mRNA at 10°C-salinity 10 ppt, 10°C-salinity 35 ppt, 15°C-salinity 10 ppt, 15°C-salinity 35 ppt was 6.7-fold, 5.0-fold, 4.0-fold and 2.6-fold higher than that of the control, respectively. Under a same low temperature (10°C or 15°C), the expression of *SpCRT* mRNA at salinity 10 ppt was always significantly higher than that at 35 ppt (*P*<0.05) while under a same salinity (10 ppt or 35 ppt), the abundance of *SpCRT* mRNA at 10°C was significantly higher than that at 15°C (*P*<0.05) (Fig 3C).

In short, the expression of *SpCRT* mRNA in the hepatopancreas, chela muscle and gills showed a similar pattern of obviously up-regulated when subjected to the four treatments of combined low temperature and salinity. Under a same low temperature (10°C or 15°C), the expression of *SpCRT* mRNA at lower salinity of 10 ppt was mostly significantly higher than that of at 35 ppt (*P*<0.05) while under a same salinity, the abundance of *SpCRT* mRNA at lower temperature 10°C was always significantly higher than that at 15°C (*P*<0.05).

### Total calcium levels in the hepatopancreas under low temperature stress

**Fig 4:**
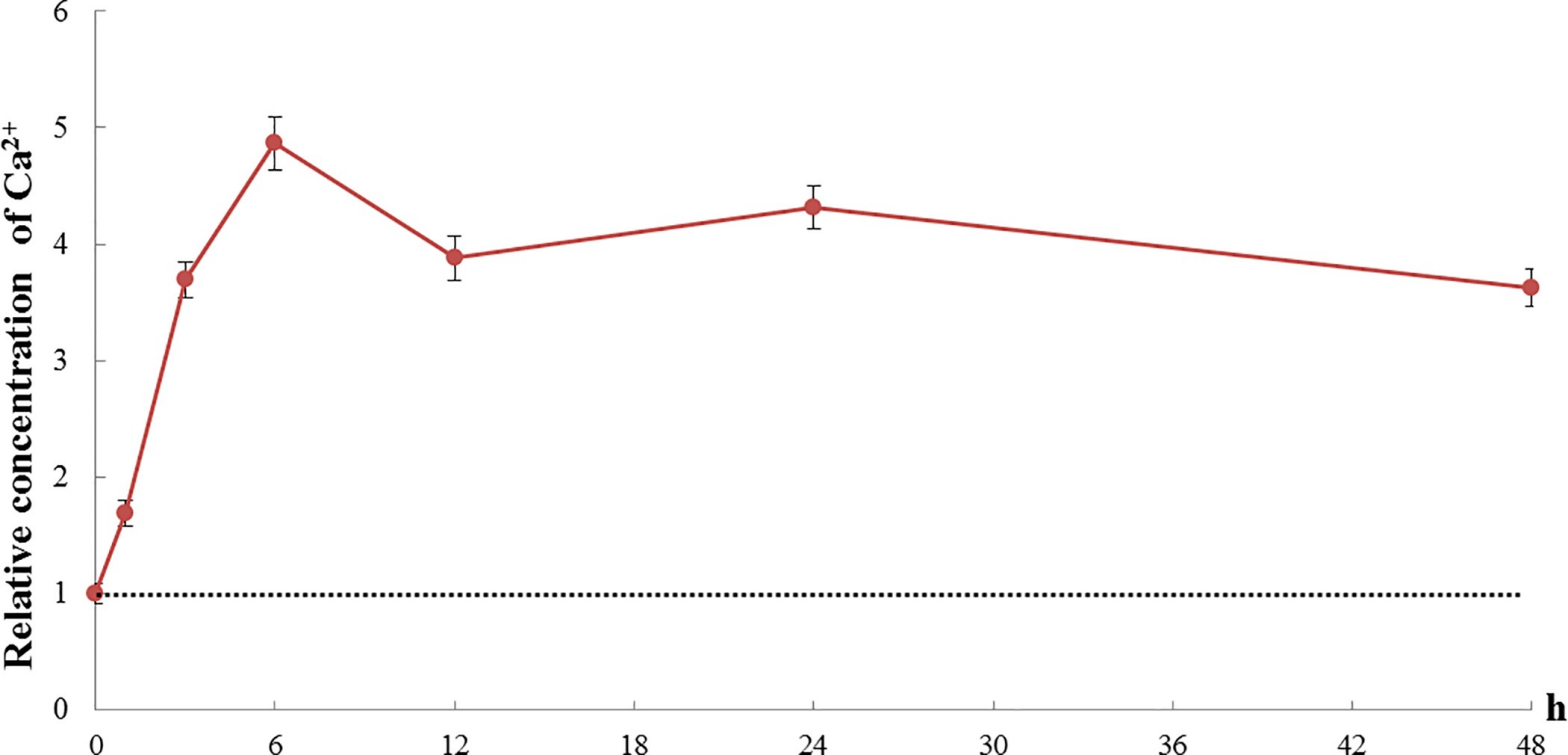
The relative concentration of Ca^2+^ in the hepatopancreas of *S*. *paramamosain* challenged by low temperature of 10°C. The dotted line represents the relative concentration of Ca^2+^ in the hepatopancreas of crabs cultured at 25°C, corresponding to the value of 1 in the Y-axis. Data are presented as mean ± standard deviation (n = 5).

When challenged with low temperature stress of 10°C, the concentration of Ca^2+^ in the hepatopancreas was significantly up-regulated and showed a pattern of rapid increase initially to reach a peak at 6 h, it then dropped back slightly at 12 h and remained relatively stable afterward (Fig 4).

### Effect of Ca^2+^ on *SpCRT* expression by *in vitro* experiment

**Fig 5:**
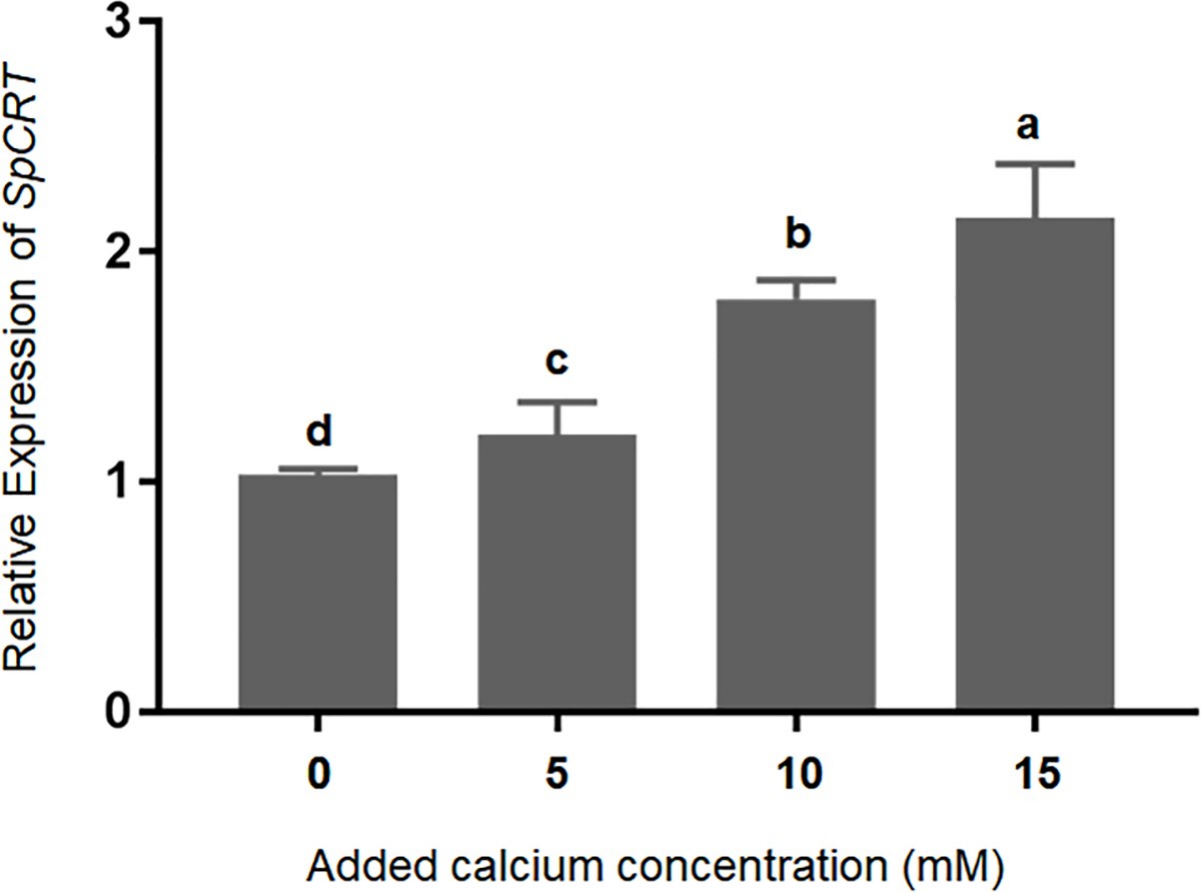
Effect of Ca^2+^ on the expressions of *SpCRT* gene in the hepatopancreas of *S*. *paramamosain* in an *in vitro* experiment. Data are presented as mean ± standard deviation (n = 5). Different letters on the top of histogram bars indicate significant differences (*P*<0.05).

The results of the *in vitro* experiment showed that with the concentration of added Ca^2+^ increased from 5 mM to 15 mM, the expression of *SpCRT* mRNA increased continuously and significantly with the highest level detected at the Ca^2+^ concentration of 15 mM, which is significantly higher than other treatments (*P*<0.05) (Fig 5).

## Discussion

When under stress of unfavorable environmental conditions, aquatic animals typically initiate various adaptive strategies, including regulating cellular metabolism and activating protection mechanisms, to maintain the homeostasis in cells, and crustaceans are no exception. It is clearly important to better understanding mechanisms underlying such adaptive strategies, particularly on molecular basis. The mud crab, *S*. *paramamosain*, as a warm water and commercially important portunid crab species, investigating the potential roles of the *CRT* gene plays on the cold adaptation of the species is obviously worthwhile and significant.

Tissue distribution detected by qRT-PCR revealed that *SpCRT* mRNA was present in all 10 tissues examined in the present study. The highest level of *SpCRT* transcript was found in the hepatopancreas. Past studies have reported high expression of *CRT* gene in livers of mammalia, fish and amphibians [29–31]. Similarly, in other crustaceans, such as *Portunus trituberculatus* and *Exopalaemon carinicauda*, *CRT* gene was also found highly expressed in the hepatopancreas [25,32]. Furthermore, the highest level of *SpCRT* transcript in the hepatopancreas was similar to the previous research of *CRT* in *S*. *paramamosain* by Xu [33]. In crustaceans, hepatopancreas is not only a major organ for Ca^2+^ storage, but also the center of reactive oxygen species (ROS) metabolism, as well as immune factors synthesis [11,33]. Therefore, it is a key organ involving in stress responses in crustaceans. The highest expression of *SpCRT* in the hepatopancreas of *S*. *paramamosain* is hence expected and consistent with its roles in stress responses of crustaceans.

The suitable temperature range for the mud crab, *S*. *paramamosain* is reportedly 18–30°C [18]. In this study, it was observed that when sub-adult mud crabs substantially reduced their activities when exposed to 15°C, and at 10°C, they were largely un-active unless touched. At 5°C, the crabs quickly became motionless and all died within 12 h. A previous study has also showed that Ca^2+^-ATPase and Ca^2+^/Mg^2+^-ATPase activities of *S*. *paramamosain* decreased sharply at 5°C [20]. Hence, it seems that 5°C is beyond the critical threshold that allows low temperature adaptation for *S*. *paramamosain*.

The present study showed that when the sub-adult *S*. *paramamosain* subjected to progressively lower temperature exposure, the pattern of *SpCRT* mRNA expression in all three tissues (hepatopancreas, chela muscle and gills) examined was very similar: The *SpCRT* expression levels at the lowest temperature 10°C were significantly higher than those crabs exposed to higher temperatures while the crabs exposed to 15°C were significantly higher than those exposed to 25 and 20°C. The *SpCRT* expression levels at 20°C were also often significantly higher than those subjected to 25°C. Hence, it is clear that the expression of *SpCRT* mRNA increased as the ambient temperature decrease, suggesting *SpCRT* gene is involved in adaptive responses to the low temperature stress. In other groups of aquatic animals, it has been also reported that when challenged by a low temperature of 4°C, the expressions of *CRT* gene in mantle and gills of the pacific oyster, *Crassostrea gigas* increased significantly as compared to that at 20°C [12]. Similarly, when the Asian seabass, *Lates calcarifer* suffered from low temperature stress, the expression of *CRT* in their livers also increased, which was suggested as the result of participation in the cold hardness [13].

When exposed to low temperatures, organisms commonly adopt the strategy of enhancing metabolism and use up more oxygen, leading to produce more ROS that are harmful to cells [34,35]. It has been reported that low temperature stress reduced the stability of protein in the swimming crab, *P*. *trituberculatus*, leading to albuminous degeneration [11]. CRT as a molecular chaperone is known to assist proper protein folding and configuration, and can prevent denaturation and degradation of proteins [14,24]. Therefore, it is likely that the role of CRT plays in stress responses is related to protein folding. In this research, the high expressions of *SpCRT* mRNA detected in hepatopancreas, chela muscle and gills of the crabs exposed to low temperatures are hence most likely related to its function as a molecular chaperone.

A further experiment on the expression of *SpCRT* mRNA as *S*. *paramamosain* were challenged by low temperatures in combination with salinity stress confirmed the results from the low temperature stress experiment and further showed that when under a same low temperature (10°C or 15°C), the expressions of *SpCRT* mRNA in the crab tissues generally increased significantly more under the low temperature-low salinity (salinity 10 ppt) combination than the low temperature-high salinity (salinity 35 ppt) combination. In other crustaceans, salinity stress has been reported to lead to increases in *CRT* gene expression in the freshwater prawn, *Macrobrachium rosenbergii* and the tiger prawn, *Penaeus monodon* [14,15]. In the swimming crab, *P*. *trituberculatus*, the expression of *CRT* gene under low salinity was higher than that under high salinity [11]. Moreover, the expression of another molecular chaperone, CCT (chaperon in containing the T-complex polypeptide-1), in *S*. *paramamosain* was also higher under low temperature and low salinity combination [21]. Temperature is known can change the spatial conformation of proteins while metallic ions for activation of the proteins are affected by salinity, hence these two most prominent environmental factors for marine and estuarine organisms appear to be interacted in regulating the activity of the proteins [36]. The findings of this study together clearly suggested that CRT plays an important role in adaptation of *S*. *paramamosain* to low temperature and salinity stresses. Our results also implied that the low temperature combined with low salinity is likely more harmful to the mud crab than in combination with high salinity. Therefore, in aquaculture setting, despite mud crab species are known to be well adapted to low salinity conditions [37], maintaining high salinity in winter under low temperatures may lessen the stress to the crabs.

A range of important physiology processes are known to relate to cellular Ca^2+^ concentration [38]. As a second messenger, Ca^2+^ is involved in many physiology processes, including secretion, cell division, cell proliferation and chromosome movement [39]. It has been demonstrated that various stresses, such as oxidization and low temperature, could lead to changes in Ca^2+^ concentration [40]. For example, low temperature stress has been reported to induce cells to produce the Ca^2+^ signal and related physiological processes, which improved the cellular resistance to the stress [41,42]. It has also been reported that removing mice from 30°C to 8°C, Ca^2+^ concentrations increased in both cardiac muscle and brain tissues [43]. Our results showed that Ca^2+^ concentration in the hepatopancreas of *S*. *paramamosain* increased significantly when exposed to low temperature of 10°C, which is consistent with those past studies on other species. Moreover, in the *in vitro* experiment, the expression of *SpCRT* mRNA in the hepatopancreas tissues dissected from *S*. *paramamosain* showed a clear trend of increasing with higher added Ca^2+^ concentration. The expression of *CRT* mRNA in mantle of the freshwater pearl mussel, *hyriopsis cumingii*, was reportedly also increased with Ca^2+^ concentration [44] and transcriptional up-regulation of *CRT* promoter was shown in Hela cells with ionomycin [45,46]. These past studies and our results together suggested that Ca^2+^ plays an important role in the regulation of *CRT* gene. In fact, in this study, it was shown that low temperature exposure at 10°C induced significant Ca^2+^ concentration increase in the hepatopancreas, which in turn could induce significant up-regulation of *SpCRT* mRNA. It suggests that Ca^2+^ probably plays a major role in signaling in low temperature adaption, which may activate not only *CRT*, but other genes related with cold adaptation.

## Conclusions

In summary, the results of present study suggest that CRT gene may plays an important role in low temperature and salinity stress responses of *S*. *paramamosain*: That is, low temperature stress induces the up-regulation of CRT gene expression via Ca^2+^ signaling, which possibly also activates other molecular chaperon, in order to maintain relevant physiological processes to achieve cold adaption.
